# Perioperative Hypothermia—A Narrative Review

**DOI:** 10.3390/ijerph18168749

**Published:** 2021-08-19

**Authors:** Simon Rauch, Clemens Miller, Anselm Bräuer, Bernd Wallner, Matthias Bock, Peter Paal

**Affiliations:** 1 Department of Anaesthesiology and Intensive Care Medicine, “F. Tappeiner” Hospital, 39012 Merano, Italy; matthias.bock@sabes.it; 2 Institute of Mountain Emergency Medicine, Eurac Research, 39100 Bolzano, Italy; 3 Department of Anaesthesiology, University Medical Centre Goettingen, 37075 Goettingen, Germany; clemens.miller@med.uni-goettingen.de (C.M.); anselm.braeuer@med.uni-goettingen.de (A.B.); 4 Department of Anaesthesiology and Intensive Care Medicine, Medical University of Innsbruck, 6020 Innsbruck, Austria; bernd.wallner@tirol-kliniken.at; 5 Department of Anaesthesiology, Perioperative Medicine and Intensive Care Medicine, Paracelsus Medical University, 5020 Salzburg, Austria; 6 Department of Anaesthesiology and Intensive Care Medicine, Hospitallers Brothers Hospital, Paracelsus Medical University, 5010 Salzburg, Austria; peter.paal@bbsalz.at

**Keywords:** hypothermia, perioperative hypothermia, body temperature regulation, perioperative care, quality of care, surgery

## Abstract

Unintentional hypothermia (core temperature < 36 °C) is a common side effect in patients undergoing surgery. Several patient-centred and external factors, e.g., drugs, comorbidities, trauma, environmental temperature, type of anaesthesia, as well as extent and duration of surgery, influence core temperature. Perioperative hypothermia has negative effects on coagulation, blood loss and transfusion requirements, metabolization of drugs, surgical site infections, and discharge from the post-anaesthesia care unit. Therefore, active temperature management is required in the pre-, intra-, and postoperative period to diminish the risks of perioperative hypothermia. Temperature measurement should be done with accurate and continuous probes. Perioperative temperature management includes a bundle of warming tools adapted to individual needs and local circumstances. Warming blankets and mattresses as well as the administration of properly warmed infusions via dedicated devices are important for this purpose. Temperature management should follow checklists and be individualized to the patient’s requirements and the local possibilities.

## 1. Introduction

Inadvertent perioperative hypothermia, defined as a drop in core temperature to <36 °C, can cause several complications. Even mild hypothermia increases the incidence of wound infection [1,2], post-operative ischaemic myocardial events [3], and blood loss during surgery [4,5], and it prolongs post-operative recovery [6,7]. Thus, providing and maintaining normothermia in the perioperative period is important for optimal surgical results as well as for patient safety and satisfaction. The incidence of perioperative hypothermia varies widely and ranges from 4% to more than 70% [8,9]. During the past two decades, awareness for the risks associated with perioperative hypothermia has increased among anaesthesiologists and surgeons. However, there is still room for further improvement [10]. In this narrative review, we described the physiology of thermoregulation, changes in thermoregulation induced by anaesthesia and surgery, consequences of perioperative hypothermia, means of core temperature measurement, and temperature management in the perioperative setting. This article is intended for all the disciplines involved in perioperative care, in particular for anaesthesiologists and surgeons. It aims at explaining the mechanisms of perioperative hypothermia and emphasising the importance of its prevention, giving concrete advice on how to do.

## 2. Physiology of Thermoregulation

Body temperature is tightly regulated in the core compartment (head and trunk). Core temperature varies by about 1 °C according to circadian rhythm and menstrual cycle. During physiological conditions, core temperature remains stable within a window of a few tenths of a degree [11]. Peripheral tissues, primarily arms and legs, act as a thermal buffer, and their temperature fluctuates considerably. For instance, in hospitalised patients exposed to ambient temperature, peripheral tissues are usually 2 to 4 °C cooler than the core [12,13].

Core temperature is tightly regulated by keeping a balance between heat gain and heat loss. Heat is generated in all body cells mainly by aerobic metabolism. The metabolic rate, and therefore heat generation at rest, is highest in the heart, kidneys, brain, and liver [14], while during exercise, most heat is produced in the skeletal muscle [15]. In an adult, the basal metabolic rate, i.e., the amount of energy per unit of time that a person requires to keep the body functioning at rest, is approximately 1 kcal/kg/h (=1.16 J/s), corresponding to 1500–1800 kcal/day (=6280–7536 kJ/d). Heat production can be increased to 600% above the basal rate by physical activity and shivering [16]. Heat loss mainly occurs via conduction (transfer of heat from the body to an object that is in direct contact), convection (transfer of heat to the air surrounding the skin), radiation (transfer of heat via infrared waves), and evaporation (transfer of heat by evaporation of water from the skin or a wound).

Thermoregulation relies on three major components, i.e., (i) temperature sensing, (ii) central temperature regulation, and (iii) efferent responses. Temperature is sensed throughout the body by thermoreceptors residing in the skin, liver, skeletal muscles, and in the hypothalamus and other parts of the central nervous system [17]. Afferent signals from these temperature sensors are conveyed to the brain mainly via tracks in the anterior spinal cord. Central thermoregulation involves the spinal cord, the brain, and in particular, the hypothalamus. The hypothalamus processes signals from the thermoreceptors and the effectors in order to keep the core temperature to its set point. The efferent response consists of autonomic and behavioural components. The primary autonomic defences in response to cold are arteriolar vasoconstriction in the fingers and toes (regulating skin blood flow and thereby heat loss to the environment [18]) and shivering [19]. Non-shivering thermogenesis via activation of brown fat by an uncoupling protein is used in preference to shivering in infants but plays a minor role in the acute thermoregulatory defence in adults [20,21]. Behavioural responses, e.g., dressing appropriately for the ambient temperature, moving briskly in a cold environment, and seeking a warm, dry, and wind-sheltered environment [22], help the autonomic system maintain core temperature.

## 3. Changes Induced by Anaesthesia and Surgery

All three periods of the perioperative setting (pre-, intra-, and post-anaesthetic period) influence the core temperature. Thus, proper temperature management starts with the patient still on the ward.

### 3.1. Pre-Anaesthetic Period

Patients with a pre-existing low core temperature before arriving in the operating room are at higher risk for remaining hypothermic intra- and postoperatively [23]. Risk factors for a pre-existing low core temperature include older age [24], low body mass index [25], and diseases, such as diabetic neuropathy [26], paraplegia, or severe hypothyroidism [27]. Emergency patients, e.g., with multiple trauma, are often accidentally hypothermic (<35 °C) on hospital admission [28,29].

Several medications can influence core temperature. For instance, antipsychotic drugs (both first and second generation) can reduce temperature [30], while antidepressants (in particular, tricyclic antidepressants) increase core temperature [31]. In particular, drugs used for preoperative anxiolysis can influence core temperature. Benzodiazepines can decrease core temperature in a concentration-dependent matter [32,33] similar to clonidine [34] and opioids [35]. Anticholinergics oppose the drop in core temperature associated with benzodiazepines [36]. Table 1 displays the influence of commonly used preoperative medications on core temperature.

During transport from the ward to the operating room, patients usually wear hospital gowns and are often covered with only a thin blanket. During this transport, heat loss can be considerable. Patients may activate thermoregulatory cutaneous vasoconstriction to maintain normal core temperature. This exposure to cold may lead to clinically relevant cooling of peripheral body regions and a temperature gradient between the core and the periphery. Induction of anaesthesia reduces the threshold for autonomic thermoregulatory responses and induces vasodilatation, which will result in the redistribution of heat from the core to the periphery of the body, thereby causing perioperative hypothermia.

### 3.2. Intra-Anaesthetic Period

**Figure 1:**
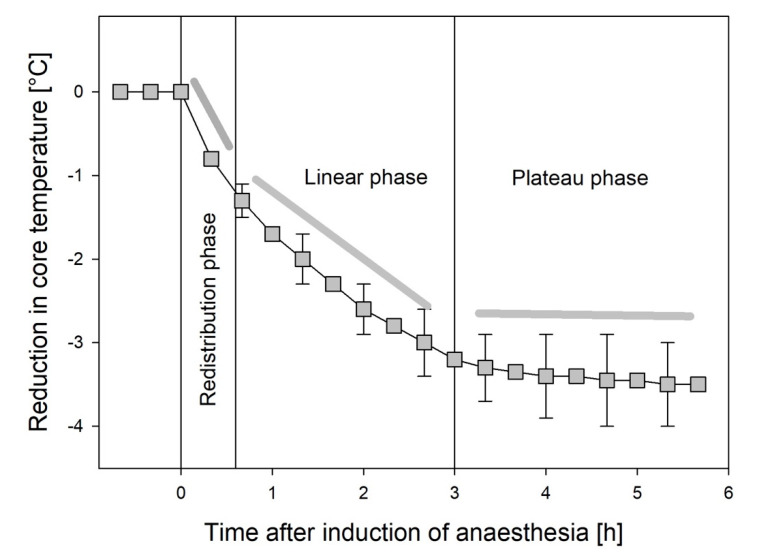
Influence of induction of general anaesthesia on core temperature. The redistribution phase, linear phase, and plateau phase can be seen. The figure shows the mean values and standard deviation (redrawn with modifications from [54]).

Hypothermia during the intra-anaesthetic period develops with a characteristic pattern and can be subdivided into three phases: redistribution, linear, and plateau (Figure 1) [37].

Redistribution of heat is the main cause of perioperative hypothermia after induction of anaesthesia, but the reduced heat generation contributes to a further decrease in core temperature. Independently of the type of anaesthesia, anaesthetics impair the autonomic thermoregulatory control because they reduce vasoconstriction and shivering thresholds [11].

Hypnotic drugs for general anaesthesia inhibit the thermoregulatory system. The hypothalamus and the spinal cord are affected by volatile anaesthetics primarily in a non-linear, concentration-dependent manner [38]. Propofol reduces core temperature in a linear, concentration-dependent way [39]. Opioids attenuate thermoregulation concentration-dependently, too, but differ in the incidence of postoperative shivering [40]. Ketamine seems to have the least influence on thermoregulation because it maintains the peripheral vascular tone and therefore limits the magnitude of blood redistribution [41]. Muscle relaxants do not pass the blood–brain barrier and therefore have no effect on thermoregulation. Drugs used for anaesthesia decrease the thermoregulatory vasoconstriction threshold in a concentration-dependent manner to around 34.5 °C [42].

The extent of temperature drop during heat redistribution from the core to the periphery after induction of general anaesthesia depends on several factors. Body morphology and the haemodynamic status of the patient play a role. For instance, redistribution is faster with higher cardiac output or peripheral vasodilatation. The most important factor is the temperature of the periphery before anaesthesia induction. The lower the temperature gradient between the core and the periphery, the lower the redistribution of heat and the lower the drop in core temperature. Leaner, smaller patients with higher blood loss cool more strongly and more quickly [43].

About an hour after induction of anaesthesia, the temperature decrease slows down and becomes more linear. While redistribution is less important in this phase, heat loss by radiation and convection prevail. Metabolic rate is reduced by about one-third that of baseline [37].

This linear phase of core temperature drop lasts about two hours and ends when the threshold of autonomous thermoregulation is reached at around 34.5 °C. The degree of the shift in the threshold for thermoregulatory defence mechanisms depends on the concentration of the anaesthetic agents administered. Vasoconstriction can subsequently be (re-)activated and transforms the thermal state of the body into a plateau phase. If the patient is actively warmed, core temperature may rise again [44]. However, further phases of redistribution can occur, e.g., after opening an arterial cross-clamp or a tourniquet.

In contrast to general anaesthesia, neuraxial anaesthesia does not impair heat production but likewise causes heat redistribution by vasodilatation in the caudad part of the body and impairs thermoregulation at the level of the spinal cord [45]. On the one hand, a patient is exposed to the cold when bare skinned for the administration of neuraxial anaesthesia. This lowers the body temperature before surgery and therefore activates the physiologic thermoregulatory vasoconstriction. On the other hand, the administered drugs prevent most of the neural activity of the caudad body and lead to a redistribution of blood similar to that in general anaesthesia. Due to its more rapid onset, hypothermia occurs more quickly in spinal than in epidural anaesthesia [46]. Furthermore, the extent of hypothermia is directly dependent on the height of the blockade [47]. A combination of neuraxial and general anaesthesia potentiates the risk of perioperative hypothermia by overlapping the effects of redistribution and vasodilatation [48]. It is assumed that extended peripheral nerve blocks, for example, of both lower limbs, will have a similar sized effect on the redistribution of heat.

Drugs commonly used for sedation have the same effect as those used in general anaesthesia. However, the concentration dependency explains the lesser effect on thermoregulation and body temperature with lower doses of sedatives. The effect of dexmedetomidine on the thermoregulatory thresholds is comparable to that of propofol [49].

In addition to the anaesthetic procedures, surgery itself has its own effects on the risk for perioperative hypothermia. First, as the temperature of the environment is key for mammals to maintain their body temperature, the temperature of the operating room should not fall below 21 °C [50]. The relationship between cutaneous heat loss and room temperature is linear [27]. Second, preparation with disinfection of large areas may lower the skin temperature. Third, large, exposed operating sites contribute to heat loss. Fourth, the insufflation of cold gases, e.g., for laparoscopy or the administration of cold irrigation fluids, e.g., for transurethral prostatic resection, can significantly lower the body temperature [51]. Fifth, deflation of tourniquets leads to a second redistribution [52]. In summary, a team approach between surgery and anaesthesia to minimise pauses in active warming therapy and to minimize loss of heat through the explained mechanisms is absolutely imperative. This applies not only for the direct effects as described but also for the indirect effects of surgery, such as the administration of intravenous fluids for substitution of fluid losses if they are inadequately warmed [53].

### 3.3. Post-Anaesthetic Period

Thermal discomfort and shivering are common complaints of patients in the post-anaesthetic phase. This underlines the importance of continuing optimal thermal management also after surgery.

## 4. Consequences of Perioperative Hypothermia

Perioperative hypothermia can affect various organ systems and has important consequences on patient outcome (Table 2).

### 4.1. Consequences on Pharmacology of Anaesthetic Drugs

Hypothermia can alter the pharmacokinetics of drugs. Hypothermia impairs the activity of enzymes, thereby decreasing and slowing the metabolism and prolonging the action of various drugs used to induce or maintain anaesthesia. Moreover, in hypothermia, the body redistributes blood from the intestine, the extremities, the kidneys, and the liver towards vital organs, which decreases the intravascular volume of distribution of various drugs. In addition, with a decrease in core body temperature, the partial pressure of carbon dioxide rises, resulting in lower pH. Depending on their pKa and acid-base status, drugs are more or less ionized when a pH shift occurs, which influences the volume of distribution [75].

Propofol is one of the intravenous drugs most frequently used for anaesthesia induction and maintenance. Propofol has a protein binding of 98% under physiological conditions and is largely metabolized in the liver. A decrease in core temperature results in an increase in plasma propofol concentration largely as a result of reduced hepatic blood flow [56]. Perioperative hypothermia also influences the potency of volatile anaesthetics by decreasing the minimum alveolar concentration (MAC) of sevoflurane and isoflurane by 5% per 1 °C drop in core temperature. Additionally, tissue solubility of volatile anaesthetics rises in hypothermia, resulting in a delayed emergence from anaesthesia [76]. The concentration of fentanyl, an opioid widely used in anaesthesia, rises by about 5% for every 1 °C decrease in the core temperature [55]. Hypothermia also influences the action of muscle relaxants by changing the distribution and/or the rate of metabolism and excretion of the drug. A reduction in core body temperature by two degrees Celsius may double the duration of the neuromuscular blockade [77].

Altogether, perioperative hypothermia is associated with delayed emergence from anaesthesia [56].

### 4.2. Hypothermia-Associated Coagulopathy

Hypothermia causes a significant impairment in plasmatic coagulation. Like other enzymes, coagulation factors require an optimal temperature range to function properly. Hypothermia reduces enzyme capacity, diminishing enzyme activity and leading to coagulopathy. The associated increased blood loss reduces the quantity of clotting factors, which further worsens blood loss. The inhibition of thrombin generation, the suppression of the thrombin burst, and fibrinogen synthesis cause a clinically significant risk for major bleeding at a core temperature < 36 °C [63,66,67]. Alterations in plasmatic coagulation are frequently missed in the clinical setting, as coagulation laboratory studies (including prothrombin time, partial thromboplastin time, and activated clotting time) are usually performed after warming blood to a temperature of 37 °C [59,61]. Rotational thromboelastometry, a point-of-care diagnostic tool, can be performed without warming blood and therefore reflects hypothermia-induced coagulation impairment with greater accuracy [58,60,62].

In addition to the effect on humoral coagulation, hypothermia affects the number and functionality of platelets. Hypothermia causes a sequestration of platelets in both the portal circulation, the liver, and the spleen as well as margination of platelets. This may lead to thrombocytopenia with a maximum decline in platelet count between 25 °C and 30 °C [65]. The hypothermia-associated thrombocytopenia is reversible as normal body temperature is restored. Hypothermia causes a reversible impairment of platelet aggregation by inhibiting the release of thromboxane A2, which plays a crucial role in platelet activation and aggregation [64]. A systematic review of major surgery cases concluded that even mild hypothermia (<1 °C) substantially increased blood loss by 16% and increased the relative risk for transfusion by 22% [4].

Hypothermic patients can also become hypercoagulable as a result of various changes in the coagulation and vascular system, such as increased viscosity, haemoconcentration, and activation of the inflammatory cascade, comparable to the effects of disseminated intravascular coagulation in septic shock patients [57].

### 4.3. Blood Loss and Transfusion Requirement

According to a meta-analysis, even mild hypothermia significantly increases blood loss [4]. A core temperature reduction of only 1 °C, for instance, was associated with an increase in the bleeding incidence (+16%) and the transfusion requirement (+22%). The risk of transfusion was significantly increased with the duration and severity of perioperative hypothermia. Even a core temperature <37 °C was associated with an increased need for blood transfusion [44]. This is important, especially in orthopaedic surgery, where lesions of small osseous vessels that cannot be coagulated mainly contribute to perioperative blood loss [67]. Active body-surface warming and maintenance of normothermia can reduce blood transfusions (odds ratio 0.6) in non-cardiac surgery [68].

### 4.4. Surgical Site Infections and Complications

Surgical site infection poses a significant risk for the postoperative outcome of patients and is among the leading causes of nosocomial infections in surgical patients [70]. Even mild perioperative hypothermia proved to be a significant and independent risk factor for surgical wound infections, with a relative risk of 6.3 [71]. Perioperative hypothermia was associated with an increased incidence of infectious complications even after a follow-up of eight weeks [5]. Hypothermia-associated surgical site infections may increase the length and overall cost of the postoperative in-hospital stay [74]. A recent metanalysis found that active body-surface warming reduces wound infections with an odds ratio of 0.3 (95% CI, 0.2–0.7) after non-cardiac surgery [68]. Perioperative hypothermia affects the immune system, and consequently the host defence against pathogens, in various ways. One detrimental effect of hypothermia is vasoconstriction. The resulting reduction in blood flow to the surgical site can impair tissue oxygenation. Tissue hypoxia impairs wound healing by altering the protein metabolism and can result in wound dehiscence [57]. Moreover, the reduced oxygen supply diminishes the oxidative immune defence mechanism used by neutrophils, for which sufficient molecular oxygen supply is needed [69,73]. Furthermore, hypothermia diminishes activation of the innate immune systems, the T-cell-mediated host defence, and targeted antibody production [72]. Hypothermia leads to a decrease in the motility of various cell types in the immune system, including platelets and macrophages [72].

### 4.5. Delayed Discharge from the Post-Anaesthesia Care Unit

The consequences of perioperative hypothermia all add up to delayed discharge from the post-anaesthesia care unit. Hypothermic patients after major abdominal surgery required approximately 40 min longer to reach fitness for discharge from the post-anaesthesia care unit even when return to normothermia was not a criterion [7]. Duration of recovery was about 90 min longer in these patients than in normothermic patients when a core temperature >36 °C was required for post-anaesthesia care unit discharge. [6,7]. This increases perioperative costs [6].

## 5. Temperature Monitoring in the Perioperative Setting

Clinicians caring for perioperative patients mainly rely on core temperature in daily routine; the recording of peripheral temperatures is reserved for specific clinical questions or scientific purposes. Skin temperature strongly depends on vasomotor tone and varies throughout various surface areas. Therefore, the site and the technique of temperature measurement are important for clinical interpretation. Mean body temperature reflects the total heat content and is calculated according to the equation: mean body temperature = 0.87 core temperature + 0.13 skin temperature [78]. The temperature in the pulmonary artery reflects the golden standard for core temperature but is seldom obtainable.

These considerations are important for the interpretation of temperature measurements: the site where the measurement is taken is usually more important than the method itself, as nearly all devices accurately monitor the temperature of the surrounding tissue. A bias (accuracy) <0.5 °C of an estimation for core temperature is generally acceptable for clinical purposes [79]. Highly perfused anatomic areas are most suitable for core temperature measurement and include the distal third of the oesophagus adjacent to the left atrium, the tympanic membrane, and the nasal pharynx. The temperature in the rectum or the bladder reflects rapid changes in core temperature with significant delay as compared to the golden standard in the pulmonary artery. This is because, with fast cooling or rewarming, feces in the rectum and urine in the bladder may cause a delay in convergence of the measured temperature as compared to the real core temperature. Due to the rapid changes in temperature in the perioperative setting, bladder and rectal temperature may not be ideal measurement sites, and when these sites are used, the delay in measurement should be considered [79,80].

Due to the level of perfusion of the adjacent structures, the correct placement of an oesophageal temperature probe is crucial for obtaining recordings of high accuracy and precision. The target position for the tip of the probe is the lower third of the oesophagus. In an adult patient of average size, this corresponds to a distance of about 40 cm from the incisors. The insertion depth of the oesophageal probe can also be determined by laying the probe on the patient. This approach is particularly useful in patients who are not of average size [81].

Clinicians must therefore verify the position of the temperature probe in the distal third of the oesophagus [81]. The temperature of adjacent devices, like endotracheal tubes, airway gases, gastric tubes, may influence measurements obtained with nasopharyngeal probes. A depth of 10–20 cm past the nares is the correct position for these devices in order to provide results comparable to those obtained with oesophageal probes [81,82].

Temperature measured on the tympanic membrane correlates well with the hypothalamic temperature, as the common carotid artery supplies both areas [79]. There are two measurement methods: tympanic probes usually equipped with thermocouples or infrared thermometry. Disposable tympanic probes are sometimes difficult to insert, as the aural canal is several centimetres long and not always straight. The patient has to verify correct placement of the probe. The risk of tympanic perforation is often mentioned although it is negligible in daily practice. Many advise against infrared thermometry of the tympanic membrane because the method has potential biases: cerumen in the aural canal might interfere, and more importantly, the thermometer might measure the temperature of the aural canal and not of the tympanum [79]. Infrared thermometers equipped with sensor technology for reduction of these confounding factors, however, produce accurate measurements with high precision even as compared to the measurements obtained with a pulmonary artery catheter [80]. Infrared thermometers measuring the forehead skin temperature are gaining importance, especially during the current COVID-19 pandemic. Unfortunately, the skin temperature of the forehead is prone to radiation and is therefore highly influenced by ambient air temperature. These devices are suitable for screening a vast population but not for a clinical situation requiring high accuracy and precision.

Zero-heat-flux thermometers provide a non-invasive method for estimating tissue temperatures. When applied to the skin, these devices create perfect thermal insulation. The temperature of the region of the skin covered by the insulator will therefore represent the core temperature after a period of equilibration. Zero-heat-flux systems consist of a flex circuit close to the skin that registers both heat-flux from the skin and skin temperature. A second device, a thermometer, is located outside the insulator. A heater guarantees that the temperature of both devices is identical. Thus, after the period of equilibration, the temperature of the external thermometer reflects the temperature of the deeper tissue under the insulator. In patients scheduled for abdominal surgery, zero-heat-flux thermometry of the forehead skin provided promising results during phases with slow temperature variations [83]. A systematic review of perioperative temperature measurements using zero-heat-flux thermometers reported large limitations among the trials included [84]. One aspect was the heterogeneity of the patients enrolled: the systematic review analysed a trial on noncardiac surgery, cardiac surgery, paediatric surgery, as well as patients recovering in intensive care units. Therefore, the authors downgraded the GRADE evidence quality to moderate. The large interval of limit of agreement from −0.93 to 0.98 °C was another important finding, leading to the conclusion that use of zero-heat-flux thermometers might not be indicated if a temperature change of less than 1 °C is clinically important. Further studies are necessary to define the clinical conditions in which zero-heat-flux thermometry is suitable.

## 6. Temperature Management in the Perioperative Period

Several national [85,86] and international guidelines [87] for perioperative temperature management have been published. In general, many recommendations are similar because all guidelines are based on the same available studies. However, some differences exist between these guidelines. The most important difference is the question of which patients should receive active perioperative temperature management.

Some guidelines, like the guidelines from the American Society of PeriAnesthesia Nurses (ASPAN) [86], list the patient groups that are at high risk for perioperative hypothermia. The guidelines also mention patients at highest risk for the development of hypothermia-related complications, like perioperative myocardial ischemia [3] and surgical site infections [1,2]. Active prewarming is restricted to these high-risk groups.

In our opinion and also according to other guidelines [87], these checklists are not sensible. The goal of these checklists is to determine which patients do not require active perioperative temperature management and where money for a forced-air warming blanket can be saved. This is misleading because every patient undergoing surgery with general or spinal anaesthesia lasting more than 30 min is at risk for perioperative hypothermia and its complications [87]. Low risk does not mean no risk, as such a checklist might suggest. The time needed for risk stratification is probably more expensive than the use of an active warming method like forced-air warming. Last but not least, hypothermia-related complications, like surgical site infections, are expensive. For instance, a surgical site infection may entail costs of € 2000 [85] to € 20,000 [88]. The avoidance of only one surgical site infection is worth 400 to 4000 warming blankets. Moreover, maintaining perioperative normothermia optimizes perioperative workflow [6]. Therefore, the National Institute for Health and Clinical Excellence (NICE) stated that active temperature management is cost-effective in all patients even if the patients are young and have a low risk for hypothermia and hypothermia-related complications [85].

**Figure 2:**
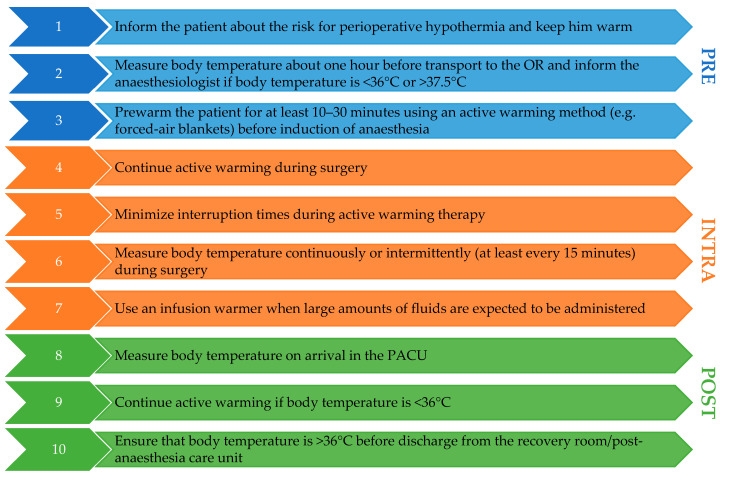
Ten essential points to prevent perioperative hypothermia for surgery lasting >30 min. Blue boxes refer to the pre-anaesthetic period, orange boxes to the intra-anaesthetic period, and green boxes to the post-anaesthetic period. OR: operating room; PACU: post-anaesthesia care unit.

We recommend that every patient undergoing surgery with general or spinal anaesthesia lasting longer than 30 min should have active thermal management. In general, the recommendations are to measure core temperature, to prewarm the patients actively before induction of anaesthesia, to warm the patients during anaesthesia, and to use fluid warming when larger amounts of fluids are used. Using this approach, postoperative hypothermia rates of less than 15% are possible [89,90]. However, it seems reasonable to pay detailed attention in order to prevent money from being spent on warming equipment without obtaining the desired benefit for the patients. Figure 2 outlines 10 essential points to prevent perioperative hypothermia for surgery lasting >30 min.

### 6.1. Active Prewarming before Induction of Anaesthesia

After being exposed to cold ambient air in the preoperative period, nearly all patients arrive in the operating room with peripheral vasoconstriction and cold peripheral tissues. Thermoregulation keeps the core of the body warm in most of these patients. However, a small percentage of the patients are already hypothermic on arrival in the operating room [91,92,93]. Active prewarming may rewarm the cold periphery of a patient, thus reducing redistribution of heat after induction of anaesthesia and reducing the initial drop in core temperature after induction of anaesthesia. To realize the maximal benefit from active prewarming, the following points are helpful:

- **Start active prewarming as soon as possible** [94]. In daily practice, the time available for prewarming is very limited. Most hospitals do not accept time delays that are avoidable because the time in the operating room is so expensive [95]. This means that active prewarming must be commenced immediately after greeting the patient. All other activities before inducing anaesthesia can be performed alongside warming. This includes sign in, monitoring the patient, and inserting intravenous, arterial, or epidural catheters. The time needed for these essential activities is also the time that is available for active prewarming [96]. Dedicated holding areas are helpful in improving the perioperative work-flow. Properly designed, these holding areas can also be cost-effective.
- **Do not skip active prewarming to save time.** Do not skip prewarming because there is not much time for prewarming. Ten minutes of active prewarming can have a huge effect and can make a big difference in the incidence of hypothermia [97].
- **Use a warming blanket.** In general, the same warming blanket should be used for active prewarming and intraoperative warming. There is no need to pay for two different warming blankets. While intraoperative warming therapy is possible with many different blankets, it is advisable to use a blanket that covers the largest part of the body surface that would otherwise be exposed to the cold. The result is the largest change in the thermal balance and the highest efficacy [96]. If a small upper-body cover is used, it can be placed lengthwise on the patient for prewarming and be placed over the legs while a thoracic epidural catheter is inserted.
- **Use the highest temperature setting recommended by the manufacturer.** The heat transfer generated by forced-air warming blankets depends on several factors. One factor is the mean temperature gradient between the warming blanket and the skin [98]. There is no rationale for reducing this temperature gradient and reducing the efficacy of forced-air warming by using lower temperatures than recommended.

### 6.2. Active Warming during Anaesthesia

During induction of anaesthesia, active warming should be continued. There is no need to stop warming for intubation or placement of arterial or central venous lines or gastric tubes [6]. Only when inserting a bladder catheter does the blanket have to be put aside for a few minutes. There is also no need to stop active warming during washing and draping, as there is no evidence that this might increase the risk of infection [99]. Nevertheless, there is clear evidence that long interruption times in active warming therapy increase the risk for hypothermia [89]. It also makes sense to insulate the parts of the body surface that cannot be actively warmed because insulation can reduce heat loss by 30% [100,101].

### 6.3. Infusion Warming

In general, infusion warming is less important than warming of the body surface. It makes sense to use an infusion warmer only when large amounts fluids are expected to be used. If an infusion warming device is used, it should be used from the start of the procedure. A short tubing after the heat exchanger of the infusion warmer or placement of the tubing under the forced-air warming blanket prevents the fluid from cooling again while it gets from the heat exchanger to the patient.

If infusion warming devices are not available, the use of prewarmed infusions from a warming cabinet is also possible and effective [102].

### 6.4. Further Possibilities

Although this approach permits the hypothermia rate to be reduced dramatically, some patients will still develop hypothermia. This may be due to long positioning times with the use of an extension table or because the body surface available for active warming is not large enough to achieve a good heat balance. Additional measures are possible and helpful to diminish this rate further in these high-risk patients:

- Use a second forced-air warming blanket, for example, to warm the patient’s legs in addition to the upper body.
- Use an additional heating blanket under the patient’s back.
- Use extended prewarming.

Other options, like the use of water mattress garments or intravascular heat exchanging catheters, are also possible and very effective but extremely expensive [103]. However, the question is: is it really cost effective to spend more than € 100 on warming equipment alone for one single patient?

## 7. Pending Studies

A prospective randomized multicentre trial (*n* = 5100) will allocate patients aged 45 years and older to standard thermal management, with active rewarming starting only when core temperature drops to 35.5 °C or to aggressive thermal management consisting of 30 min prewarming prior to anaesthesia induction and intraoperative warming with a target core temperature of 37–37.5 °C; IV fluids will be warmed to body temperature. The primary outcome is a composite of myocardial injury after non-cardiac surgery, non-fatal cardiac arrest, and all-cause mortality. Secondary outcome parameters are deep or organ-space surgical site infection, intraoperative transfusion requirements, duration of hospitalization, and readmission rates. The estimated study completion date is December 2022 (ClinicalTrials.gov Identifier: NCT03111875).

## 8. Conclusions

Perioperative hypothermia is common and associated with increased blood loss, transfusion requirements, incidence of wound infections, length of stay in the post-anaesthesia care unit, and costs. Temperature management in the pre-, intra-, and postoperative period is crucial to diminish the risks of perioperative hypothermia. Awareness of perioperative hypothermia, temperature measurement with accurate and continuous probes, and active body surface warming before induction of anaesthesia and during surgery are essential to maintain perioperative normothermia and diminish the risks for perioperative hypothermia. Temperature management should follow checklists and be individualized to the patient’s requirements and according to the local possibilities.

## Figures and Tables

**Table 1:** Overview of common preoperative medications and their influence on core temperature.

Class of Drugs	Influence on Core Temperature	References
Antidepressants (particularly tricyclic antidepressants)	Increase	[31]
Antipsychotics (both first and second generation)	Decrease	[30]
Benzodiazepines	Decrease	[32,33]
Clonidine	Decrease	[34]
Opioids	Decrease	[35]
Anticholinergics	Oppose the temperature decrease from benzodiazepines	[36]

**Table 2:** Main consequences of perioperative hypothermia.

Effects of Perioperative Hypothermia	References
Alteration of pharmacokinetics of drugs used for anaesthesia	[55,56]
Coagulopathy	[4,57,58,59,60,61,62,63,64,65,66,67]
Increased blood loss	[4,44,57,68]
Increased transfusion requirements	[4,44,57,68]
Increased risk for surgical site infections	[5,57,69,70,71,72,73,74]
Delayed discharge from the post-anaesthesia care unit	[6,7]

## Data Availability

Not applicable.
